# Loss of protein tyrosine phosphatase non-receptor type 2 reduces IL-4-driven alternative macrophage activation

**DOI:** 10.1038/s41385-021-00441-3

**Published:** 2021-08-21

**Authors:** Marianne R. Spalinger, Meli’sa Crawford, Sarah D. Bobardt, Jiang Li, Anica Sayoc-Becerra, Alina N. Santos, Ali Shawki, Pritha Chatterjee, Meera G. Nair, Declan F. McCole

**Affiliations:** Division of Biomedical Sciences, University of California Riverside, Riverside, CA USA

## Abstract

Macrophages are a heterogeneous population of innate immune cells that are often divided into two major subsets: classically activated, typically pro-inflammatory (M1) macrophages that mediate host defense, and alternatively activated, tolerance-inducing (M2) macrophages that exert homeostatic and tissue-regenerative functions. Disturbed macrophage function/differentiation results either in inadequate, excessive immune activation or in a failure to induce efficient protective immune responses against pathogens. Loss-of-function variants in protein tyrosine phosphatase non-receptor type 2 (*PTPN2*) are associated with chronic inflammatory disorders, but the effect of macrophage-intrinsic PTPN2 loss is still poorly understood. Here we report that PTPN2-deficient macrophages fail to acquire an alternatively activated/M2 phenotype. This was the consequence of reduced IL-6 receptor expression and a failure to induce IL-4 receptor in response to IL-6, resulting in an inability to respond to the key M2-inducing cytokine IL-4. Ultimately, failure to adequately respond to IL-6 and IL-4 resulted in increased levels of M1 macrophage marker expression in vitro and exacerbated lung inflammation upon infection with *Nippostrongylus brasiliensis* in vivo. These results demonstrate that PTPN2 loss interferes with the ability of macrophages to adequately respond to inflammatory stimuli and might explain the increased susceptibility of *PTPN2* loss-of-function carriers to developing inflammatory diseases.

## Introduction

Macrophages are cells of the innate immune system, which are present in all tissues and exert key innate immune functions such as the removal of cell debris and invading pathogens.^1^ Besides their remarkably high phagocytic potential, macrophages contribute to immune responses via antigen presentation and the secretion of cytokines and chemokines, thereby contributing to the activation of both, adaptive and innate immune cells.^1,2^ Despite these common traits, macrophages are a heterogeneous cell population and possess the ability to polarize into phenotypically and functionally different subsets depending on the environmental cues they encounter during activation.^3^ The two main categories of macrophage polarization phenotypes are discriminated on whether the cells exhibit a generally pro-inflammatory (M1-like) or a more anti-inflammatory/suppressive (M2-like) phenotype. Classical activation of immature macrophages by IFN-γ and/or lipopolysaccharide (LPS) results in inflammation-promoting M1 macrophages that trigger T-helper cell (Th)1 and Th17 dominated, mainly inflammatory immune responses, whereas alternative activation of macrophages with interleukin (IL)-4, IL-13, glucocorticoids, or IL-10 generates anti-inflammatory or type 2 immune response-provoking M2 macrophages.^4,5^ However, classification in M1 and M2 macrophages is somewhat outdated as several sub-forms of M2 macrophages have been described and macrophage phenotypes are highly plastic with macrophages being able to differentiate along a continuum of phenotypes with classically activated, pro-inflammatory (hereafter referred to as M1) macrophages and alternatively activated, immune-suppressive (here termed M2) macrophages representing two differentiation extremes.^5,6^ Furthermore, macrophage responses to activation and maturation cues also depend on the origin of the macrophages, i.e., whether they are fetal-derived, tissue-resident or monocyte-derived.^5,6^

The factors that govern macrophage activation and polarization are diverse, and the fate of a specific cell is often the result of a cascade of signaling events. Among the typical cytokines that have been used to provoke an M2-like macrophage phenotype, IL-4 has been widely used in in vitro studies.^4^ Of interest, however, IL-6, which has potent pro-inflammatory effects,^7^ also promotes the development of M2-like macrophages by enhancing the expression of the alpha subunit of the IL-4 receptor (IL-4Rα),^8^ thus enabling efficient anti-inflammatory macrophage induction.

Over a decade ago, genome-wide association studies identified loss-of-function variants in the gene locus encoding protein tyrosine phosphatase non-receptor type 2 (PTPN2) as a risk factor for several autoimmune and inflammatory disorders, including rheumatoid arthritis, type 1 diabetes, celiac disease, metabolic syndrome, and inflammatory bowel diseases.^9,10^ Over the past decade, several publications investigated the role of PTPN2 in T cells to show how T cell specific PTPN2 loss facilitates the development of inflammation via promotion of interferon-gamma (IFN- γ)/type 1 dominated immune activation and loss of tissue tolerance.^11–13^ Our group has demonstrated important roles for PTPN2 in maintaining normal functions of the intestinal epithelial barrier, and its loss resulted in exaggerated IFN-γ-induced epithelial barrier defects.^14,15^ Additional studies with mice lacking PTPN2 in hepatocytes revealed important functions of PTPN2 in prevention of oxidative stress.^16^ In contrast, the role of PTPN2 in innate immune cells is less studied. We have recently shown that loss of PTPN2 in myeloid cells results in increased susceptibility to experimental colitis, while protecting from colitis-associated cancer in an inflammasome-dependent manner,^17^ but the molecular consequences of PTPN2 deletion in myeloid cells, and especially in macrophages, are still not well understood.

On a molecular level, PTPN2 deactivates Janus kinases (JAKs) and signal transducer and activator of transcription (STAT) molecules,^11,18^ with a prominent effect on STAT1 and STAT3,^13,19^ which play a pivotal role in macrophage polarization towards an M1 or an M2 phenotype, respectively.^20–22^ Notably, we have observed reduced M2 differentiation in PTPN2-deficient/knockdown macrophages even though these macrophages produce elevated levels of IL-6.^23^ Given this conundrum, the aim of our study was to elucidate the mechanisms that prevent PTPN2-deficient/knockdown macrophages from polarizing into an anti-inflammatory, M2 phenotype. Furthermore, we investigated the molecular response of PTPN2-deficient macrophages to IL-6 and IL-4, two cytokines that synergistically promote M2 polarization.

## Results

### Loss of PTPN2 compromises IL-6 induced signaling

**Fig. 1:**
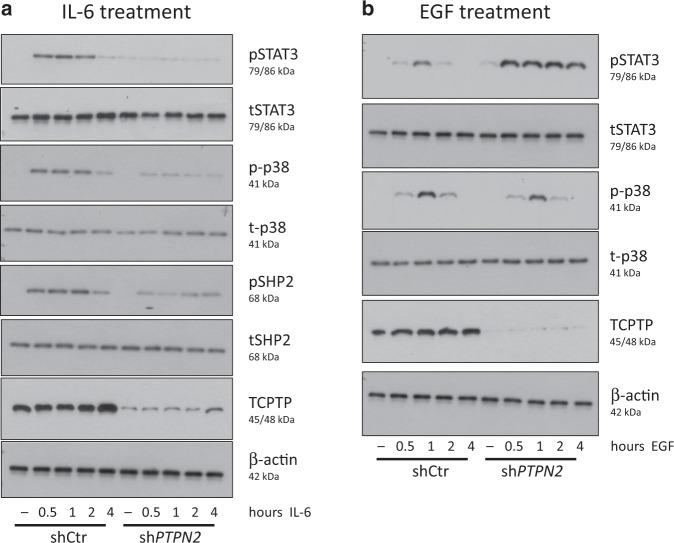
Knockdown of PTPN2 abolished the intracellular response to IL-6. THP-1 cells expressing either non-targeting control (shCtr) or *PTPN2*-specific (shPTPN2) shRNA were differentiated into macrophages and subsequently treated for the indicated time with **a** 20 ng/ml IL-6 or **b** 50 ng/ml EGF. Depicted are representative Western blot images for the indicated proteins from five independent experiments (*n* = 5).

To understand why PTPN2-deficient macrophages fail to develop into M2 macrophages despite their high intrinsic levels of IL-6, we investigated the molecular response of PTPN2-deficient macrophages to treatment with IL-6. PMA-differentiated THP-1 macrophages expressing non-targeting control (shCtr) or PTPN2-specific (shPTPN2) shRNA constructs were treated over time with IL-6 and phosphorylation of the IL-6-induced signaling molecules, STAT3, p38, and SHP-2 analyzed. While in Ctr THP-1 cells, IL-6 induced fast and sustained phosphorylation of all three analyzed IL-6-induced signaling molecules; pSTAT3, p-p38 and pSHP-2 were only marginally activated in PTPN2-knockdown macrophages upon treatment with IL-6 (Fig. 1a), even though PTPN2-knockdown macrophages exhibited increased basal STAT3 phosphorylation levels. Notably, the absence of activation of these signaling molecules was not due to a general inability of PTPN2-knockdown macrophages to react to external stimuli, or a general failure to activate these pathways, as EGF treatment resulted in induction of p38 in both, PTPN2-knockdown and PTPN2-competent THP-1 cells and, consistent with previous reports, STAT3 activation was further enhanced in PTPN2-knockdown macrophages upon EGF treatment (Fig. 1b). Similar findings were obtained in bone marrow-derived macrophages (BMDM) from *Ptpn2*-LysMCre mice (Supplementary Fig. S1), which lack PTPN2 in myeloid cells. This indicates that PTPN2-compromised macrophages are less responsive to IL-6 and fail to activate intracellular signaling cascades upon IL-6 treatment.

### Reduced IL-6Rα expression in PTPN2-deficient macrophages

**Fig. 2:**
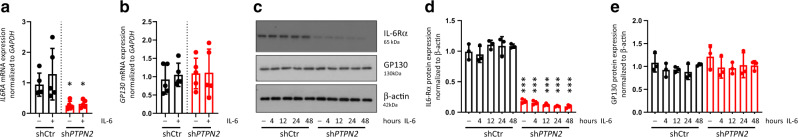
Reduced IL-6Rα expression in PTPN2-knockdown macrophages. THP-1 cells expressing non-targeting control (shCtr) or *PTPN2*-specific (shPTPN2) shRNA were differentiated into macrophages and treated for 24 h (A + B) or the indicated time (C–E) with 20 ng/ml IL-6 and analyzed for **a** *IL6RA* and **b** *GP130* mRNA levels, as well as **c**–**e** IL-6Rα and GP130 protein levels. * = *p* < 0.05, *** = *p* < 0.001 relative to the same condition in shCtr cells, ANOVA with Dunnett’s multiple comparisons test; representative results from one out of three independent experiments with *n* = 5 (A + B) or *n* = 3 (C–E) independent replicates.

Having shown that PTPN2-knockdown/deficient macrophages are less responsive to IL6 treatment, we next analyzed whether those cells express normal levels of the IL-6 receptor. Consistent with our previous observations using a NanoString multiplex mRNA screening assay,^23^ we detected reduced mRNA expression of the IL-6R alpha chain (IL-6Rα), *IL6RA*, in PTPN2-knockdown macrophages, while mRNA expression of the IL-6R beta chain, *GP130* was unaltered (Fig. 2a, b). These findings were fully confirmed on a protein level, where we also detected reduced levels of IL-6Rα, but no change in GP130 protein levels (Fig. 2c–e). Again, similar results were obtained with bone marrow-derived macrophages from *Ptpn2*-LysMCre mice, which expressed less IL-6Rα protein when compared to BMDMs from their PTPN2^fl/fl^ littermates, while levels of gp130 were unaltered (Supplementary Fig. S2). These findings show reduced expression of the IL-6 binding subunit of the IL-6 receptor (IL-6Rα) upon loss or knockdown of PTPN2, which explains the disrupted responsiveness to IL-6 of PTPN2-compromised macrophages.

### Failure to induce IL-4R upon IL-6 treatment

**Fig. 3:**
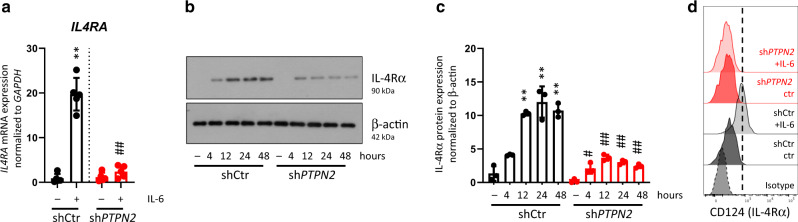
PTPN2-knockdown macrophages fail to induce IL-4Rα upon treatment with IL-6. THP-1 cells expressing non-targeting control (shCtr) or *PTPN2*-specific (shPTPN2) shRNA were differentiated into macrophages and treated for 24 h (A + D) or the indicated time (B + C) with 20 ng/ml IL-6. Depicted are **a** mRNA expression of *IL4RA* normalized to *GAPDH* and untreated shCtr cells, **b** representative Western blot images for IL-4Rα and β-actin, **c** respective densitometry analysis, and **d** representative flow cytometry histograms for surface IL-4Ra expression. Gated on life, single cells. ** = *p* < 0.01 relative to untreated control, ^##^ = *p* < 0.05 relative to IL-6 treated shCtr cells, ANOVA with Dunnett’s multiple comparisons test. Representative results from one out of three independent experiments with *n* = 5 (A + B) or *n* = 3 (C + D) independent replicates.

The M2-promoting cytokine IL-4 is sensed by IL-4R, which consists of an alpha (IL-4Rα) subunit and the common IL-2 receptor gamma chain (γc, mainly present in hematopoietic cells) or IL-13Rα1 (mainly present in non-hematopoietic cells).^24^ Notably, IL-4Rα expression is low in resting macrophages, though its expression is induced upon treatment with IL-6.^8^ In line with this, in Ctr THP-1 macrophages, IL-6 treatment resulted in a strong upregulation of *IL4RA* mRNA levels. However, and consistent with a general failure to respond to IL-6, PTPN2-knockdown macrophages only marginally up-regulated *IL4RA* upon IL-6 treatment (Fig. 3a). Western blot analyses confirmed these results on a protein level: while IL-4Rα was absent in lysates from resting THP-1 cells, IL-6 treatment resulted in strong and sustained IL-4Rα protein levels and surface expression in PTPN2-competent but not in PTPN2-knockdown macrophages (Fig. 3b–d). Again, similar effects were observed in BMDM from WT vs. *Ptpn2*-LysMCre mice (Supplementary Fig. S3).

### Reduced M2 polarization upon treatment with IL-4

**Fig. 4:**
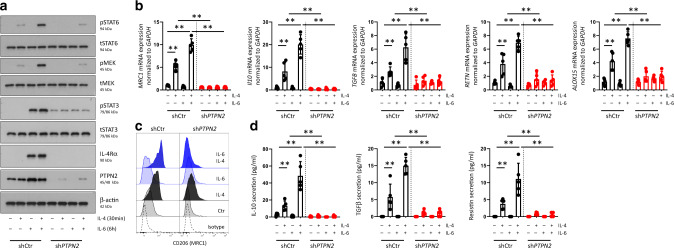
Knockdown of PTPN2 inhibits IL-6-mediated promotion of IL-4 signaling and alternative macrophage activation. THP-1 cells expressing non-targeting control (shCtr) or *PTPN2*-specific (shPTPN2) shRNA were differentiated into macrophages and treated with IL-6 for 6 h prior to treatment with IL-4 for 30 min (A) or 24 h (B–D). The graphs show **a** representative Western blot images for the indicated proteins, **b** mRNA expression of *MRC1* (encoding CD206), *IL10* and *TGFB, RETN, ALOX15;* **c** flow cytometry for CD206 surface expression (gated on live, single cells), and **d** IL-10 and TGFβ and resistin levels in the cell culture medium. ** = *p* < 0.01 relative to untreated control cells, ANOVA with Dunnetts’s multiple comparisons test. Representative results from three independent experiments with five independent replicates (*n* = 5).

In line with a failure to up-regulate IL-4Rα, *PTPN2*-knockdown THP-1 macrophages and *Ptpn2*-deficient BMDM failed to induce downstream signaling cascades, such as STAT6 and MEK phosphorylation upon treatment with IL-4 alone or after pre-treatment with IL-6 (Fig. 4a, Supplementary Fig. 4). Consistent with previous reports, IL-4 treatment induced mRNA expression of the M2 (alternatively activated) macrophage marker *MRC1* (encoding for CD206) and *IL10, TGFB, RETN*, and *ALOX15* mRNA expression in Ctr macrophages, an effect further enhanced after pre-treatment with IL-6 (Fig. 4b). However, PTPN2-knockdown macrophages failed to respond to IL-4 and IL-6 treatment, resulting in diminished expression of those regulatory molecules (Fig. 4b). Similar effects were observed on a protein level, where IL-4/IL-6 induced surface expression of CD206 and cytokine secretion of IL-10, TGFβ, and resistin, which were all diminished in PTPN2-knockdown macrophages (Fig. 4c, d). Notably, the effects of IL-4 were not dependent on basal levels of IL-6 in the culture, since adding anti-IL-6 to the medium did not affect the expression of molecules associated with M2 macrophages (Supplementary Fig. S5).

**Fig. 5:**
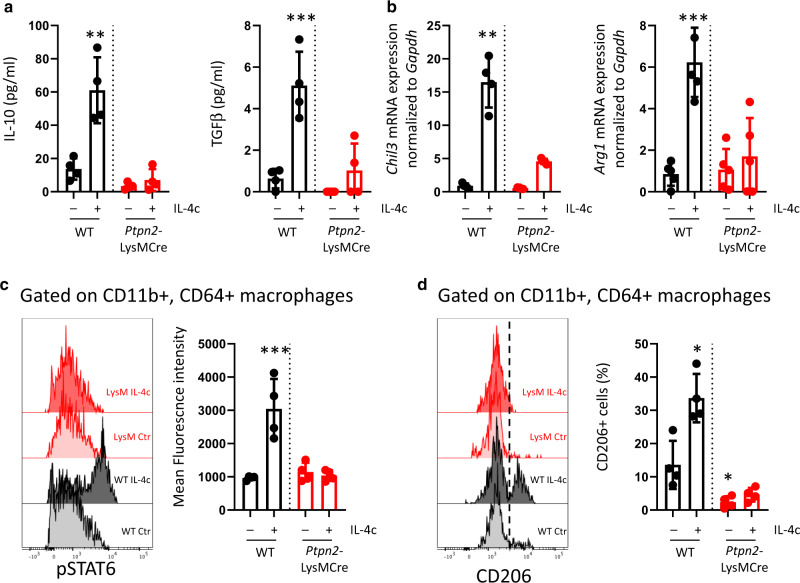
PTPN2 deficiency in myeloid cells results in loss of macrophage response to IL-4 in vivo. WT and *Ptpn2*-LysMCre mice were injected intraperitoneally with IL-4-IL-4R immune complexes and **a** peritoneal lavages analyzed for IL-10 and TGFβ after 24 h, **b** peritoneal immune cells collected after 24 h and analyzed for mRNA expression of *Chil3* and *Arg1* normalized to WT controls and *Gapdh;* **c** peritoneal immune cells collected after 2 h and STAT6 phosphorylation analyzed in peritoneal macrophages (gated as live, single, CD45+, CD11b+, Gr1−, CD64 + cells), and **d** peritoneal immune cells collected 24 h after injection and macrophages analyzed for the proportion of CD206+ cells. Gated as in **b**. * = *p* < 0.05, ** = *p* < 0.01, *** = *p* < 0.001, ANOVA with Holm-Sidak’s multiple comparisons test. Representative results from two independent experiments with 3–4 mice per group.

Likewise, IL-4 injection in vivo resulted in robust STAT6 phosphorylation in peritoneal and colonic macrophages, increased relative abundance of CD206 + macrophages, and enhanced production of IL-10 and TGFβ in the peritoneal cavity and the colon of WT mice, effects significantly reduced in *Ptpn2*-LysMCre mice (Fig. 5, Supplementary Fig. S6). Notably, and in line with our previous results,^23^ untreated *Ptpn2*-LysMCre mice harbored reduced basal levels of CD206+ macrophages in the peritoneal cavity and the colonic lamina propria (Fig. 5, Supplementary Fig. S6). Of interest, *Ptpn2*-deficient macrophages were also resistant to M2 induction with IL-10, but they did respond with upregulation of CD206 in the presence of glucocorticoids (Supplementary Fig. S7), indicating that the defect in M2 polarization is stimulus-specific and context dependent. In contrast to reduced response to M2-inducing factors and in line with our previous findings, *Ptpn2-*LysMCre mice were more susceptible to M1-induction upon treatment with IFNγ and LPS in vivo and in vitro (Supplementary Fig. S8). Notably, IL-6 slightly reduced M1-associated factors in WT cells, while it did not have an effect in cells from *Ptpn2-*LysMCre mice (Supplementary Fig. S8).

### Soluble IL-6Rα restores IL-6-induced response to IL-4 and facilitates M2 macrophage polarization in PTPN2-deficient macrophages

**Fig. 6:**
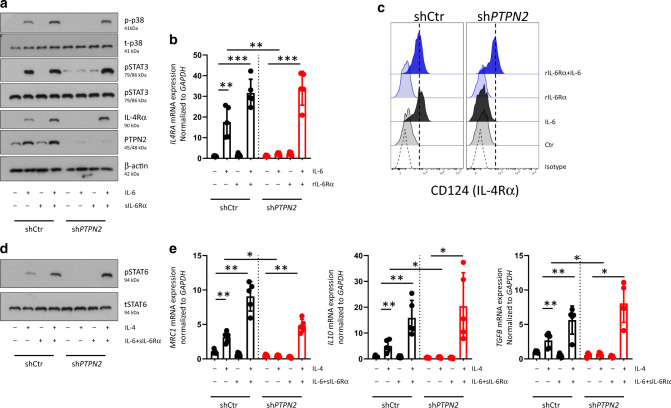
Addition of soluble IL-6Rα restores the capacity of PTPN2-knockout macrophages to respond to IL-6. **a**–**c** THP-1 cells expressing non-targeting control (shCtr) or *PTPN2*-specific (sh*PTPN2*) shRNA were differentiated into macrophages and treated with IL-6 in combination with or without recombinant sIL-6Rα for 30 min (A) or 24 h (B + C). Depicted are **a** representative Western blot pictures for the indicated proteins, **b** mRNA expression levels of *IL4A* normalized to *GAPDH* and untreated control cells, **c** flow cytometry histograms for surface expression of IL-4Rα (CD124). **d**, **e**: THP-1 cells expressing non-targeting control (shCtr) or *PTPN2*-specific (sh*PTPN2*) shRNA were differentiated into macrophages and treated with IL-6 in combination with recombinant sIL-6Rα for 6 h prior to stimulation with IL-4 for 30 min (D) or 24 h (**e**, **f**). The graphs show **d** representative Western blot images and **e** mRNA expression of *MRC1* (encoding CD206), *IL10*, and *TGFB* normalized to *GAPDH* and untreated control cells. * = *p* < 0.05, ** = *p* < 0.01, *** = *p* < 0.001, ANOVA with Dunnett’s multiple comparisons test. Representative results from three independent experiments with 5 independent replicates (*n* = 5).

Having shown that PTPN2-compromised macrophages failed to respond to IL-6, which leads to decreased levels of IL-4Rα expression, we hypothesized that restoring normal IL-6 signaling might rescue the inability of PTPN2-compromised macrophages to respond to IL-4. To address this, we employed the fact that IL-6 can bind to a soluble form of the IL-6Rα chain, sIL-6Rα, and that these IL-6-sIL-6Rα complexes then bind to and signal through GP130.^25^ Indeed, treatment with IL-6 in combination with sIL-6Rα restored the ability of PTPN2-knockdown macrophages to induce a robust intracellular IL-6 response resulting in normal levels of p38 phosphorylation and—in line with PTPN2 being a negative regulator of STAT3—enhanced levels of STAT3 phosphorylation (Fig. 6a). Furthermore, adding sIL-6Rα during IL-6 treatment resulted in normal induction of *IL4RA* mRNA and protein expression and surface localization (Fig. 6b, c). Addition of sIL-6Rα in combination with IL-6 and IL-4 also restored STAT6 phosphorylation and expression of the M2 marker *MRC1* in response to IL-4 and re-established the secretion of IL-10 and TGFβ in PTPN2-knockdown macrophages (Fig. 6d–f). Summarized, this indicates that correcting the inability of PTPN2-knockdown macrophages to respond to IL-6 restored their capacity to develop into regulatory/alternatively activated M2 macrophages.

### Loss of PTPN2 in myeloid cells excacerbates lung inflammation upon infection with *Nippostrongylus brasiliensis*

**Fig. 7:**
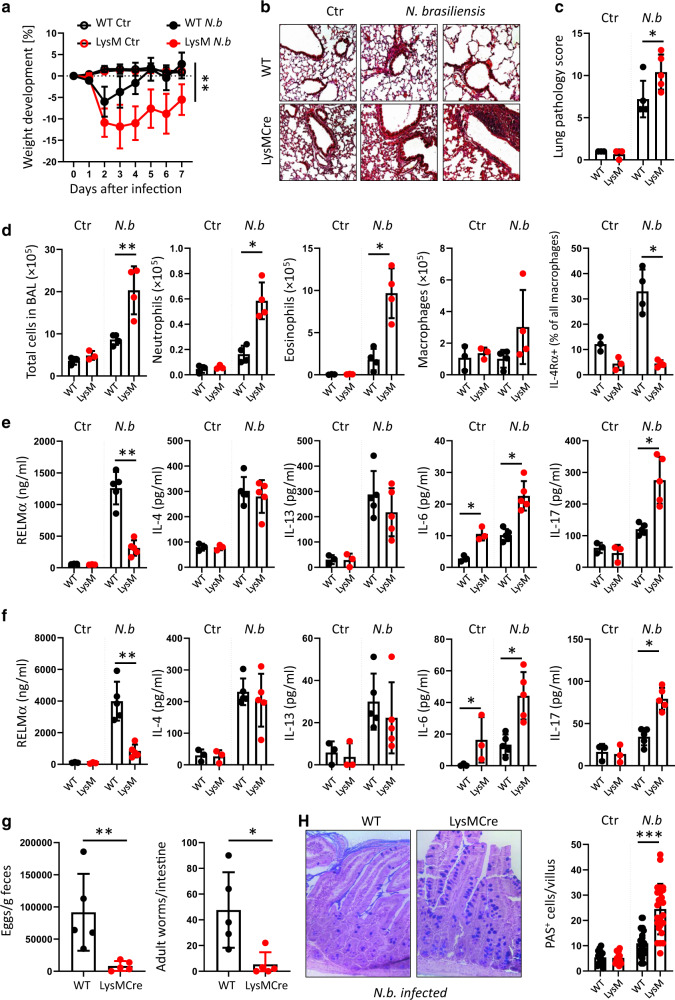
*Ptpn2*-LysMCre mice are more susceptible to *Nippostrongylus brasilensis* infection. 7-week-old *Ptpn2*-LysMCre and their WT littermates were infected with 500 *Nippostrongylus brasiliensis* L3 stage larvae. **a** Weight development post infection, **b** representative pictures of lung histology and **c** respective scoring, **d** numbers of infiltrating cell, neutrophils, eosinophils, and macrophages as well as IL-4Ra expression on macrophages in BAL fluid at day 7. **e**, **f** levels of the indicated cytokines in **e** BAL fluid and **f** small intestinal homogenate on day 7 post infection. **g** Egg and worm count in the small intestine and the feces on day 7. **h** Alciane blue-PAS staining and goblet cell counts of small intestinal sections from mice on day 7. *N.b*. = *Nippostrongylous brasiliensis* infected. Representative results from one out of two independent experiments with 4-6 mice per group, each. * = *p* < 0.05, ** = *p* < 0.01, *** = *p* < 0.001, Kruskal–Wallis with Dunn’s multiple comparisons test.

Since macrophages, and especially alternatively activated/M2 macrophages, provide protection against helminth infections and helminth-induced tissue damage,^26,27^ we next assessed whether the failure of PTPN2-compromised macrophages to develop into M2 macrophages affects the response to *Nippostrongylus brasiliensis* (*N. brasiliensis*) infection. In line with a failure to induce M2 macrophages, *Ptpn2*-LysMCre mice suffered from more pronounced *N. brasiliensis*-induced weight loss, elevated red blood cell counts in bronchio-alveolar lavages (BAL) between day 2–7 post infection, enhanced lung pathology, and an increase in infiltrating cells, including neutrophils and eosinophils in BAL 7 days post infection (Fig. 7a–d, Supplementary Figs. S9, S10a+b). Numbers of infiltrating macrophages, however were not affected, but BAL macrophages from *Ptpn2*-LysMCre mice expressed significantly reduced levels of IL-4Rα (Fig. 7d). In line with a reduced ability to produce alternatively activated macrophages, infection-induced levels of RELMα were reduced in the BAL of *Ptpn2*-LysMCre mice, while IL-4 and IL-13 were not affected and IL-6 and IL-17 were elevated (Fig. 7e). In the lungs of *N. brasiliensis*-infected *Ptpn2*-LysMCre mice, knockout of *Ptpn2* was observed in alveolar and interstitial macrophages, while eosinophils, neutrophils and dendritic cells showed minimal reduction in *Ptpn2* mRNA expression (Supplementary Fig. S11). Consistent with our in vitro findings, *Ptpn2-*deficient interstitial and alveolar macrophages showed a clear reduction of *Il4ra* and *Il6ra* mRNA expression (Supplementary Fig. S11). A similar cytokine profile as in the lung was observed in the small intestine (Fig. 7f). Interestingly, *Ptpn2*-LysMCre mice showed reduced egg and worm burden in the small intestine (Fig. 7g), which was associated with increased numbers of goblet cells (Fig. 7h). In line with our in vitro findings that addition of IL-6Rα to the cell culture medium was able to restore the ability of PTPN2-knockdown macrophages to develop into M2 macrophages, injection of IL-6Rα during infection with *N. brasiliensis* ameliorated infection-induced weight loss and lung pathology in *Ptpn2-*LysMCre mice (Supplementary Fig. S12). In line with more severe inflammation during primary infection and a general elevated number of inflammatory cells, *Ptpn2-*LysMCre mice also suffered from increased lung leukocyte infiltration upon secondary infection with *N. brasiliensis* (Supplementary Fig. S10c). Overall, this indicates that the failure of PTPN2-compromised macrophages to develop into M2 macrophages has profound in vivo consequences, resulting in decreased protection against helminth-induced lung pathology, while the overall elevated immune response in the lung and intestine led to decreased worm burden.

## Discussion

Our work demonstrates that loss of PTPN2 in macrophages resulted in impaired IL-6Rα expression and consequently a lack of responsiveness to IL-6. This ultimately rendered PTPN2-deficient/knockdown macrophages incapable of inducing IL-4Rα in response to IL-6, which impeded them from IL-4-mediated M2 polarization during inflammatory immune responses. Adequate, and temporally orchestrated macrophage activation, with an initial induction of M1 macrophages to induce protective immunity against pathogens, followed by increased of M2 macrophage induction to counteract tissue damage and promote regeneration and homeostasis, is crucial to prevent excessive and chronic immune reactions. In line with this, mice lacking *Ptpn2* in myeloid cells suffered from increased lung inflammation in response to *N. brasiliensis* infection, likely due to reduced induction of M2 macrophages to control the immune response.^26^ Notably a report by Sutherland et al demonstrated that IL-17—which is elevated in infected PTPN2-LysMCre mice—promotes worm killing in the lung,^28^ possibly explaining the reduced worm burden. PTPN2 is associated with a number of inflammatory disorders, (e.g., rheumatoid arthritis, diabetes), where pathologically high levels of IL-6,^29,30^ and altered macrophage activity have been associated with the disease.^31^ Thus, besides the well-described role of PTPN2 in the adaptive immune system,^11,13^ disturbed macrophage polarization upon loss of PTPN2 might represent an additional key factor as to why PTPN2 loss of function variants are associated with chronic inflammatory disorders,^9^ which underlines the relevance of our findings not only in the setting of helminth infections, but for a broad number of diseases.

Although PTPN2-compromised macrophages produce elevated levels of IL-6,^23,32^ here we demonstrate that PTPN2-deficient macrophages are impaired in their response to IL-6. IL-6 is often regarded as a generally pro-inflammatory molecule, and its presence is required for the development of highly inflammatory cells, such as Th17 cells, that act as drivers of pathological inflammation.^33^ However, with its capacity to induce expression of IL-4 receptor,^8^ and its effect in enhancing the capacity of macrophages to develop/polarize into alternatively activated/M2 macrophages,^21^ IL-6 also exerts anti-inflammatory effects. The failure of PTPN2-deficient macrophages to respond to IL-6 and the consequent failure to up-regulate IL-4Rα, caused the incapacity to react to IL-4, which ultimately generated a defect in M2 polarization.

We further showed that the failure of PTPN2-deficient/knockdown macrophages to induce M2-specific markers, or develop an IL-4-mediated M2 phenotype, was indeed due to an impaired response to IL-6 and in particular to the absence of proper IL-6Rα expression, since addition of soluble IL-6Rα was able to fully restore the capacity of PTPN2-deficient/knockdown macrophages to respond to IL-4 and develop into M2 macrophages. This further indicates that lack of PTPN2 does not result in a general incapacity to activate signaling cascades relevant for M2 polarization, but rather represents a selective reduction in surface expression of receptors required to deliver M2-inducing cues to the cell.

At first it seemed surprising that PTPN2-deficient/knockdown macrophages show diminished activation of STAT3, given that PTPN2 acts as a negative regulator of this particular signaling pathway.^11,13^ However, as shown by treatment with EGF, this lack of a response to IL-6 was not due to an inability to induce STAT3 in general, but due to a specific incapacity to respond to IL-6. It will certainly be of great interest to further investigate the mechanisms leading to the reduced IL-6Rα expression we observed in PTPN2-deficient/knockdown macrophages. IL-6Rα has several regulatory elements, and its expression can be enhanced by STAT3. In light of increased basal STAT3 activation in PTPN2-deficient/knockdown cells the reduced IL-6Rα levels are surprising. However, there might be counter-regulatory mechanisms in response to chronically elevated basal phospho-STAT3 levels that affect IL-6Rα expression. One molecule that controls the IL-6Rα/STAT3 axis is micro-RNA (miR)34,^34^ which is also involved in the regulation of M2 macrophages.^35^ It has been suggested that PTPN2 modulates expression of certain micro RNAs,^36,37^ and thus it might be of interest to evaluate in a follow-up study whether PTPN2 loss affects the expression of miR34 and other miRNAs involved in macrophage biology.

The reduced capacity of PTPN2-defective macrophages to develop into anti-inflammatory/M2 macrophages is not only relevant within the context of inflammation, but might also play a pivotal role in the response to tumors. It has been shown that tumor-associated macrophages, which represent a distinct subset of M2 macrophages, contribute to poor prognosis and suppress efficient anti-tumor immune responses^38–40^ and might be a driving force for the failure of anti-PD-1 treatment in low immunogenic tumors.^41^ In line with this, we have previously shown that mice lacking PTPN2 in myeloid cells suffer from more severe dextran sulfate sodium-induced colitis, while at the same time they were protected from tumors in a mouse model of colitis-associated carcinoma.^17^ Depending on their polarization status, macrophages can either suppress or promote anti-tumor immune responses,^40^ and while an increased tendency to develop into pro-inflammatory M1 macrophages, or an inability to develop into M2 macrophages, can promote inflammation, alternatively activated M2 macrophages within the tumor micro-environment play an essential role in suppressing anti-tumor immune responses,^41^ thus their absence might allow for more efficient tumor clearance in mice lacking PTPN2 in myeloid cells. Therefore, the reduced ability of PTPN2-deficient/kncokdown macrophages to develop into alternatively activated (M2) macrophages during inflammation might contribute to the enhanced inflammation but reduced tumor load that we previously observed in PTPN2-LysMCre mice.^17^

In summary, we demonstrate that loss/knockdown of PTPN2 in macrophages profoundly impairs their ability to respond to IL-6 and to upregulate IL-6-induced IL-4Rα expression, which abrogates M2/anti-inflammatory macrophage polarization. These findings fundamentally contribute to the mechanistic understanding of how PTPN2 regulates innate immune cell activation and how innate immune cells shape immune responses. Given the importance of an adequate, dynamic anti-inflammatory *vs*. pro-inflammatory macrophage balance for the resolution of inflammation in various diseases—including diseases that have previously been associated with *PTPN2* loss-of-function variants—our findings not only contribute to the understanding how defective PTPN2 promotes susceptibility for inflammatory diseases, but also promotes understanding of how defects in fine-tuning macrophage differentiation contribute to pathologies and is therefore relevant for a broad number of physiological and pathophysiological events.

## Materials and methods

### Cell lines and cell culture

THP-1 cells were maintained as described^42^ and PTPN2 knockdown introduced using lentiviral particles using standard techniques.^23^ In brief, lentiviral particles were generated via transfection of HEK293T cells with non-targeting control (shCtr) or *PTPN2-*specific (sh*PTPN2*) shRNA constructs from Sigma-Aldrich (St. Louis, MO) together with a packaging (pR8.2) and an envelope encoding plasmid (pMDG.2) (gifts from Dr. R. Daniel Beauchamp, Vanderbilt University, Nashville, TN) using Effectene reagent (Qiagen, Valencia, CA). The lentiviral particle containing supernatant was collected after 24 h, filtered (0.45-μM, Millipore, Billerica, MA), and applied to THP-1 cells in the presence of polybrene (5 mg/ml). 72 h later, shRNA expressing cells were selected using puromycin (0.25 μg/mL) after which the cells were maintained in puromycin containing medium (0.25 μg/mL). *PTPN2* knockdown was confirmed by RT-PCR and Western blot (data not shown).

To generate BMDM bone marrow cells from 9 to 10 week old mice (see below) were grown for 7 days in L929 supernatant-containing culture medium as described.^43^

For macrophage differentiation, THP-1 cells were pulsed with 50 ng/ml phorbol myristate acetate (PMA; Sigma-Aldrich, St. Louis, MO) for 3 h, washed and seeded into 12 well plates. After 48 h, the medium was changed to serum-free RPMI and the cells left untreated, treated with 20 ng/ml recombinant IL-6 (Peptrotech, Rocky Hill, NJ), 50 ng/ml recombinant IL-4 (Peprotech), 10 nM dexamethasone (Sigma-Aldrich, St. Louis MO), 50 ng/ml IL-10 (Peprotech), or 50 ng/ml recombinant EGF (Peprotech) as indicated. In some experiment, recombinant IL-6Rα (1 μg/ml; BioLegend, San Diego, CA) was added to the culture medium at the same time as recombinant IL-6. For IL-6 inhibition, the culture medium was supplemented with 1 μg/ml anti-IL-6 (BioLegend).

### Protein extraction and Western blotting

To isolate proteins, cells were washed in PBS, re-suspended in RIPA buffer (150 mM NaCl, 5 mM EDTA, 50 mM Tris, 1% NP-40, 0.5% sodium deoxycholate, 0.1% SDS, supplemented with complete mini protease inhibitor cocktail from Roche, Basel, Switzerland), incubated for 30 min and sonicated for 30 s. The lysates were centrifuged at 12,000 *g* and protein-containing supernatant used for Western blotting. Equal amounts of protein were loaded on polyacrylamide gels, separated by gel-electrophoresis, and blotted onto PVDF membranes. Membranes were blocked with 3% milk and 1% BSA in wash buffer (Tris buffered saline, 0.1% Tween), incubated overnight with primary antibodies in wash buffer, washed and incubated with HRP-coupled anti-mouse, anti-rabbit or anti-goat secondary antibodies (Jackson ImmunoResearch, West Grove, PA) for 2 h. Immunoreactive proteins were detected using an enhanced chemiluminescence detection kit (Thermo Fisher Scientific) and X-ray films (Labscientific Inc., Highlands, NJ). Densitometric analyses were performed using FIJI (Image J). The used antibodies and sources are listed in Supplementary Table S1.

### RNA extraction, cDNA synthesis, and qPCR

For RNA isolation, cells were washed in ice-cold PBS and RNA isolated using the RNeasy Mini Kit (Qiagen, Venlo, NL) according to manufacturer’s instructions. RNA was transcribed into complementary DNA (cDNA) using the qScript cDNA synthesis kit from Quantabio (Beverly, MA) following the manufacturer’s instructions. iQ SYBR Green Supermix (Bio-Rad, Hercules, CA) was used for real-time quantitative PCR on a C1000 Thermal cycler equipped with a CFX96 Real-Time PCR system and the BioRad CFX Manager 3.1 Software. The PCR included an initial enzyme activation step (3 min, 95 °C) followed by 45 cycles of denaturing (95 °C, 10 s), annealing (53–60 °C, 10 s) and extending (72 °C, 10 s). Measurements were performed in triplicates and human or mouse *GAPDH/Gapdh* served as endogenous controls. Results were analyzed by the ΔΔCT method. The primers used are listed in Supplementary Table S1.

### ELISA

IL-10 and TGFβ DuoSet ELISA was obtained from R&D Systems (Minneapolis, MN) and performed according to the manufacturer’s guidelines. Resistin and RELMα ELISA was performed as described.^44^

### Flow cytometry

THP-1 macrophages and bone marrow-derived macrophages were detached using ice-cold PBS, 0.1% EDTA. Lamina propria cells, peritoneal immune cells and lung immune cells were isolated as described previously.^13,44,45^ The cells were then incubated with FcR blocking antibody (Miltenyi Biotec, Bergisch Gladbach, Germany) for 10 min and stained with anti-CD45-Pacific Blue, anti-CD3-BV650, anti-NK1.1-BV650, anti-B220-BV650, anti-CD11b-BV605, anti-CD11c-PECy7, anti-Ly6C-PerCPCy5.5, anti-F4/80-Fitc, anti-CD64-PE, anti-MHC-II-AF700, anti-CD206-PE-TexasRed, anti-pSTAT6-APC (all from BioLegend, mouse cells) anti-CD124-PE, anti-CD206-APC, (all from Biolegend, human cells) for 15–30 min. ZOMBI-NIR live dead stain (BioLegend, San Diego, CA) was used for discrimination between live and dead cells in all experiments. For pSTAT6 staining, cells were permeabilized using the FoxP3 staining kit from eBioscience. Samples were acquired on an LSRII cytometer (BD, Franklin Lakes, NJ), and analyzed using FlowJo (Tree Star, Inc. Ashland, OR). For RNA expression analysis of lung immune cell susbsets, lung homogenates were sorted on a MoFlo Asterios EQ cell sorter (Beckman Coulter Life Sciences, Krefeld, Germany).

### Mice, IL-4 treatment and Nippostrongylus brasiliensis infection

PTPN2^fl/fl^-LysMCre^+/−^ mice were provided by Prof. Michael Scharl at the Department for Gastroenterology and Hepatology of the University Hospital Zurich, Zurich, Switzerland. These mice express a loxP flanked exon 3 of the *Ptpn2* gene (*Ptpn*2^*fl/fl*^; originally from EUCOMM) and Cre under the Lysozyme promoter (LysMCre, originally obtained from the Jackson Laboratory). *Ptpn2*^*fl/fl*^-LysMCre^−^ mice (termed WT mice throughout the manuscript) were bred with *Ptpn2*^*fl/fl*^-LysMCre^+/−^ mice (referred to as KO or *Ptpn2*-LysMCre mice throughout the manuscript) to yield *Ptpn2*^*fl/fl*^-LysMCre^−^ and *Ptpn2*-LysMCre^+/−^ littermates. The mice were housed in a specific-pathogen-free facility with food and water ad libitum and 9–12 week old male and female littermates were used for all studies. Mice were matched for sex and age and allocated randomly to the experimental and control groups. Investigators were blinded thorughout the experiment. Recombinant IL-4 (Peprotech, 5 μg/mouse) was administered as a complex of two molecules of IL-4 bound by one molecule of a monoclonal anti-IL-4 antibody (11B11, BioXCell, Lebanon, NH) as described to enhance IL-4 stability and its biological half-life/availability in the body.^46,47^ For *Nippostrongylus brasiliensis* infections, mice were infected subcutaneously with 500 larval stage 3 larvae, and parasite worms were counted from the dissected small intestine, and eggs quantified microscopically in the feces. H&E staining, lung pathology assessment and PAS staining was performed as described.^44^ For secondary infection, mice were infected again with 500 stage 3 larvae 4 weeks after the first infection and lung tissue collected 7 days after the secondary infection.^48,49^ All experiments involving mice were approved by the IACUC of the University of California, Riverside (IACUC protocol number A20190032E).

### Statistical analyses

Data are presented as mean ± SD for *n* biological replicates. Animal data are presented as mean ± SD. The number of mice in each experiment are given in the figure legends. Statistical analysis was performed using ANOVA followed by Dunnett’s or Holm-Sidak post-test or Kruskal–Wallis followed by Dunn’s test for multiple comparisons as indicated in the figure legends. The experimental unit was the mouse. Sample sizes were estimated using an expected effect size of 0.25. No animals were excluded from the analyses. *P* values of <0.05 were considered significant.

## Supplementary information

Supplementary information

Supplementary Table 1

## Data Availability

All data underlying this study are presented in the figures of this manuscript and its supplementary data.
