# Supplementary material for: Loss of protein tyrosine phosphatase non-receptor type 2 reduces IL-4-driven alternative macrophage activation
Source: Mucosal Immunol. 2021 Aug 21;15(1):74–83. doi: 10.1038/s41385-021-00441-3 (PMC8732276; doi:10.1038/s41385-021-00441-3)
Supplement: Supplementary file 1 — Supplementary information [file 41385_2021_441_MOESM1_ESM.pdf]

Supplementary Figure S1

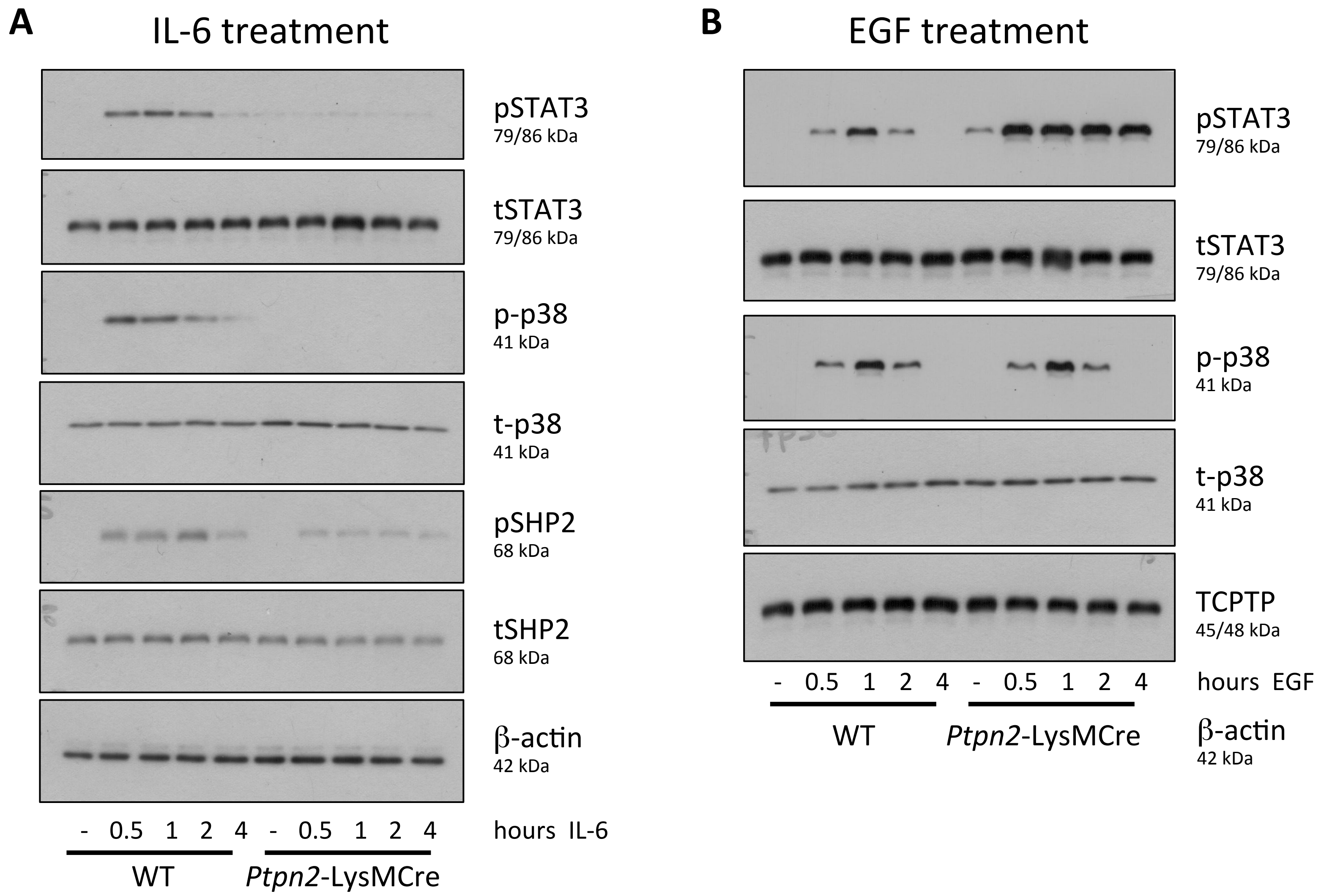

Supplementary Figure S1. Impaired response to IL-6 in PTPN2-deficient BMDM. BMDM from WT or *Ptpn2*-LysMCre mice were treated for the indicated time with IL-6 (A) or EGF (B) and analyzed for the indicated proteins. n = 5 independent repetitions. Related to main Figure 1.

Supplementary Figure S2

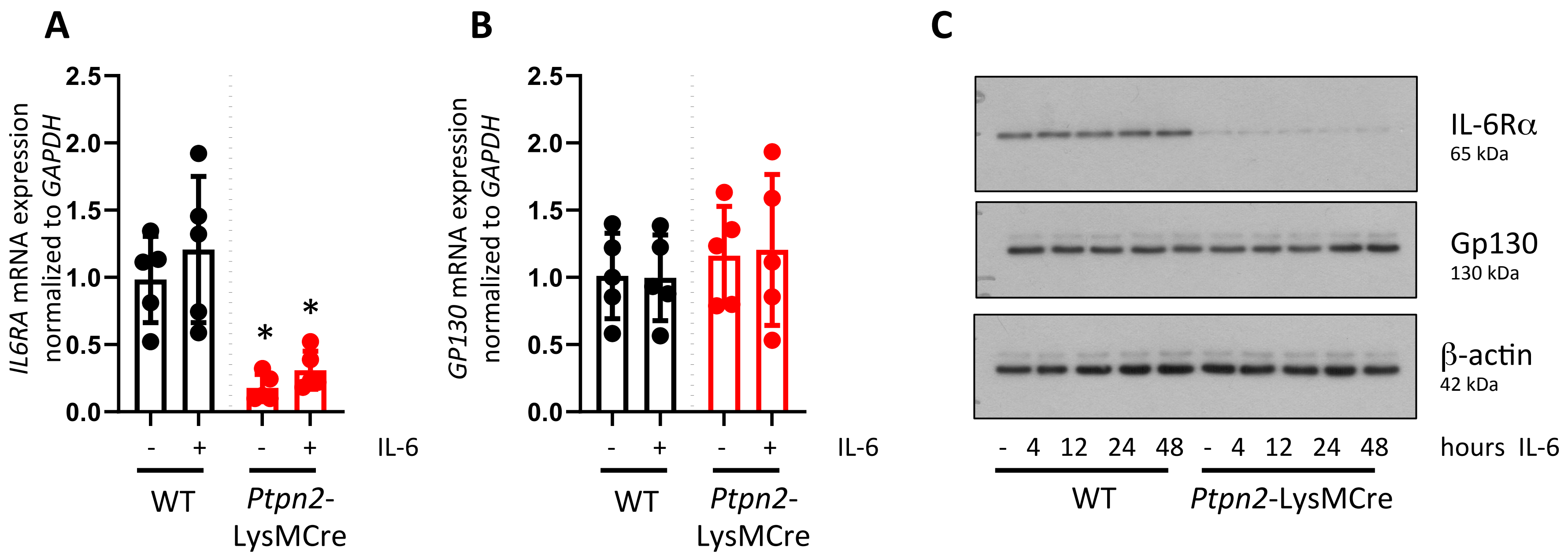

Supplementary Figure S2. Diminished IL-6Ra expression in PTPN2-deficient BMDM. BMDM from WT or *Ptpn2*-LysMCre mice were treated for 24 h (A+B) or the indicated time (C) with IL-6. mRNA expression levels of **A)** *Il6ra*, and **B)** *Gp130* normalized to *Gapdh* and untreated WT cells. **C)** Representative Western blot pictures for the indicated proteins. n = 5 independent repetitions. * = p < 0.05, ANOVA with Dunn's multiple comparisons test. Related to main Figure 2.

Supplementary Figure S3

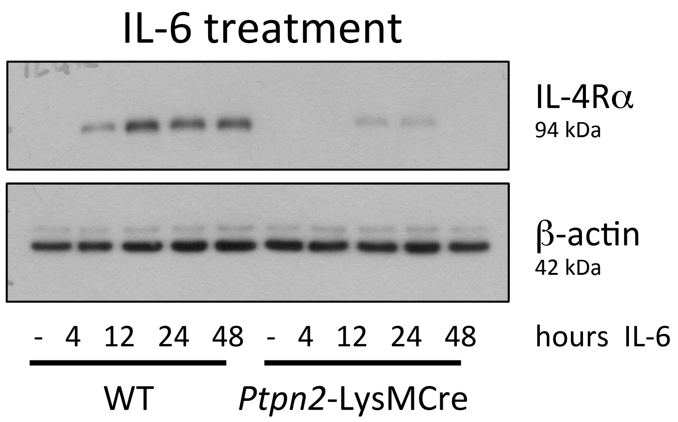

Supplementary Figure S3. Lack of IL-6-induced IL-4Ra upregulation in PTPN2-deficient BMDM. Bone marrow-derived macrophages from WT or *Ptpn2*-LysMCre mice were treated for the indicated times with IL-6 and analyzed by Western blot for IL-4Ra expression. n = 3 independent repetitions with three technical replicas each. Related to main Figure 3.

Supplementary Figure S4

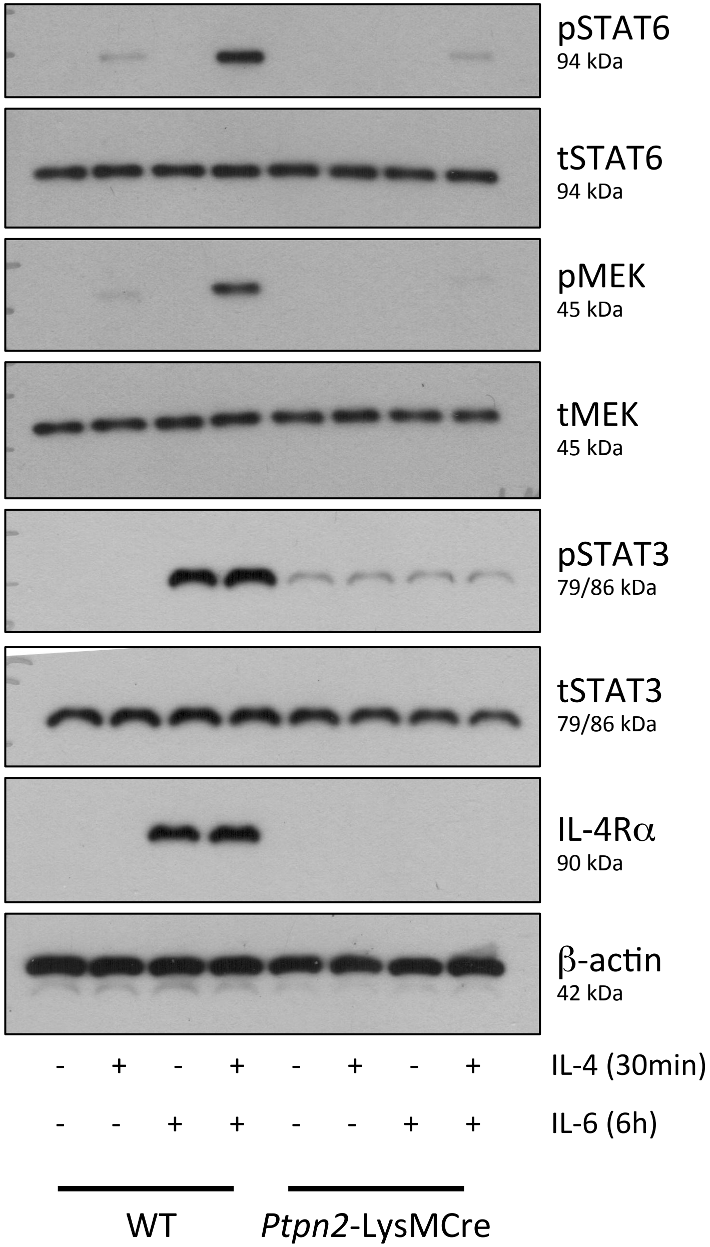

Supplementary Figure S4. PTPN2-deficient BMDM fail to react to IL-4. Bone marrow-derived macrophages from WT or *Ptpn2*-LysMCre mice were treated for 6 h with IL-6 prior to treatment with IL-4 for 30 min. and analyzed by Western blot for the indicated proteins. n = 3 independent repetitions. Related to main Figure 4.

Supplementary Figure S5

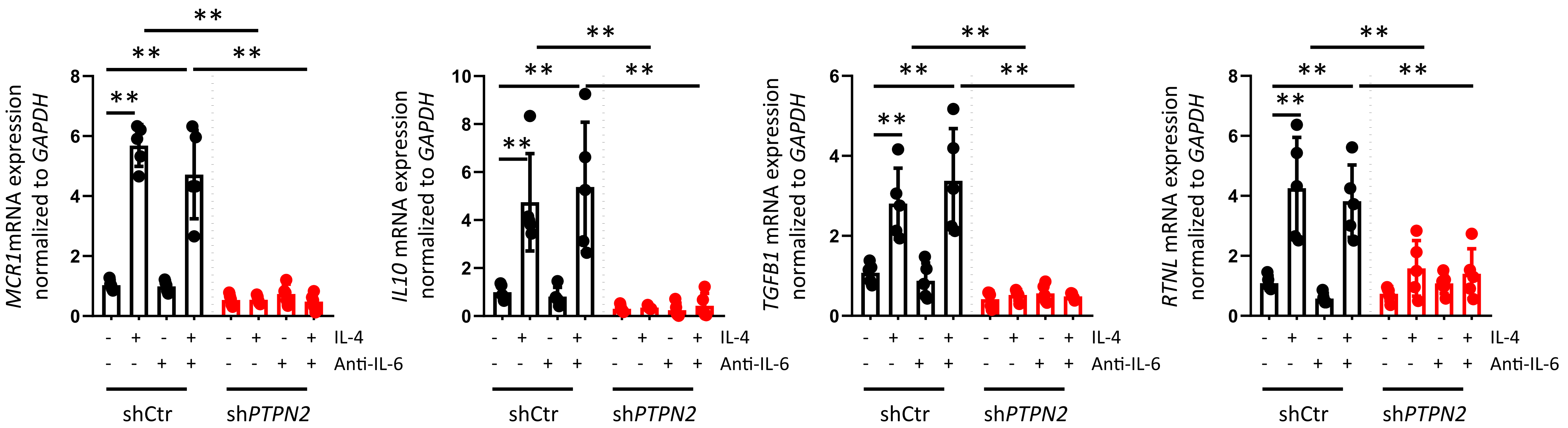

Supplementary Figure S5. Basal activation of the IL-6 pathway during IL-4 treatment is not responsible for reduced response to IL-4. THP-1 cells expressing non-targeting control (shCtr) or *PTPN2*-specific (shPTPN2) shRNA were differentiated into macrophages and treated with IL-4 in presence with anti-IL-6 for 24 h and analyzed for mRNA expression of *MRC1* (encoding CD206), *IL10* and *TGFB,* and *RTNL.* ** = p < 0.01, ANOVA with Dunnetts's multiple comparisons test. Representative results from five independent experiments (n=5).

Supplementary Figure S6

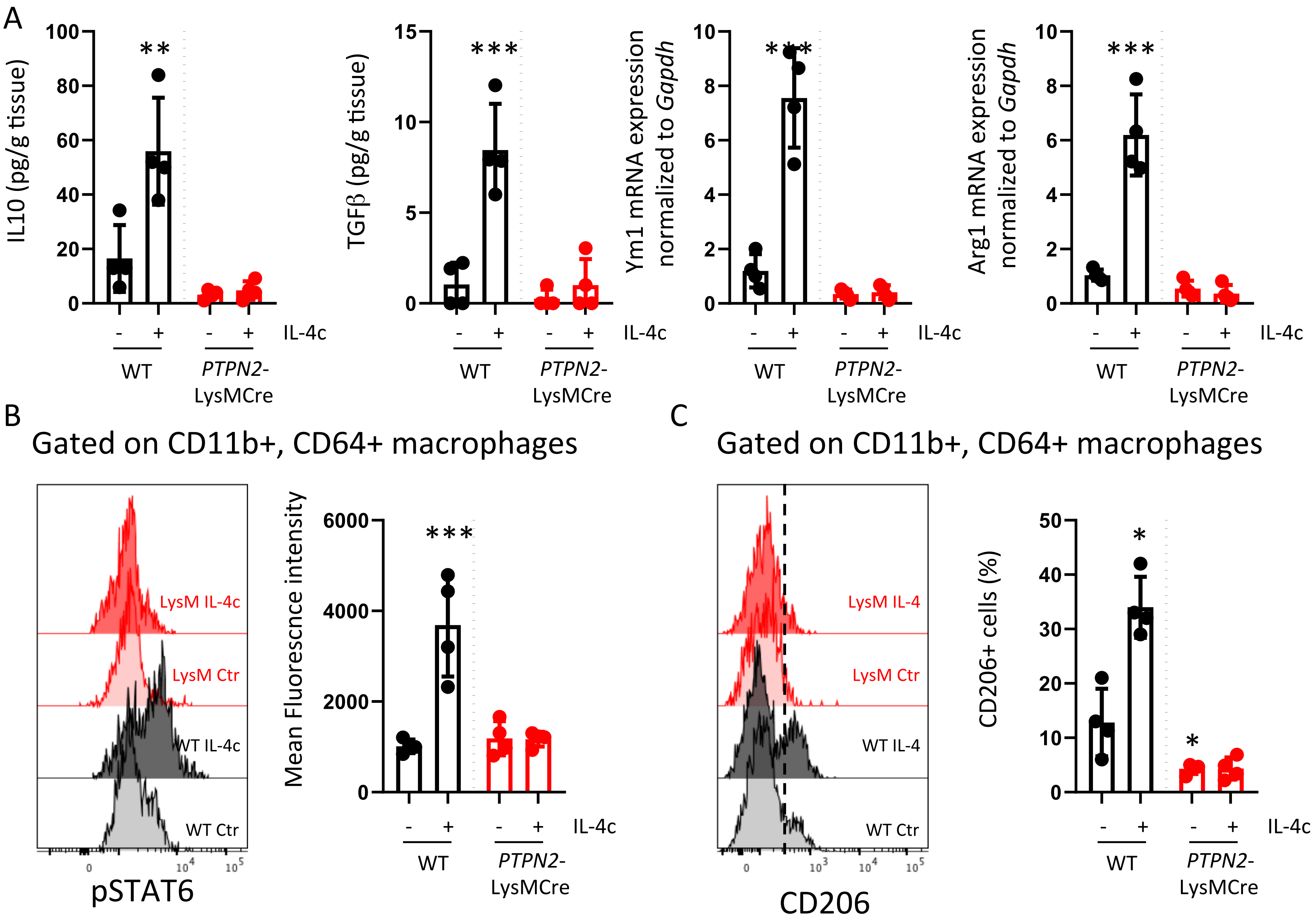

Supplementary Figure S6. PTPN2 deficiency in myeloid cells results in loss of macrophage response to IL-4 in the intestine. A) WT and *Ptpn2*-LysMCre mice were injected intraperitoneally with IL-4-IL-4R immuncomplexes and **A)** colonic tissue analyzed for IL-10 and TGFb after 24 h, **B)** colonic lamina propria immune cells collected after 2 h and STAT6 phosphorylation analyzed in colonic macrophages (gated as live, single, CD45+, CD11b+, Gr1-, CD64+ cells), **C)** colonic lamina propria immune cells were isolated 24 h after injection and macrophages analyzed for the proportion of CD206+ cells. Gated as in (B). * = $p < 0.05$, ** = $p < 0.01$, *** = $p < 0.001$, ANOVA with Holm-Sidak's multiple comparisons test. Related to main Figure 5. Representative results from one out of two independent experiments with 3-5 mice/group.

Supplementary Figure S7

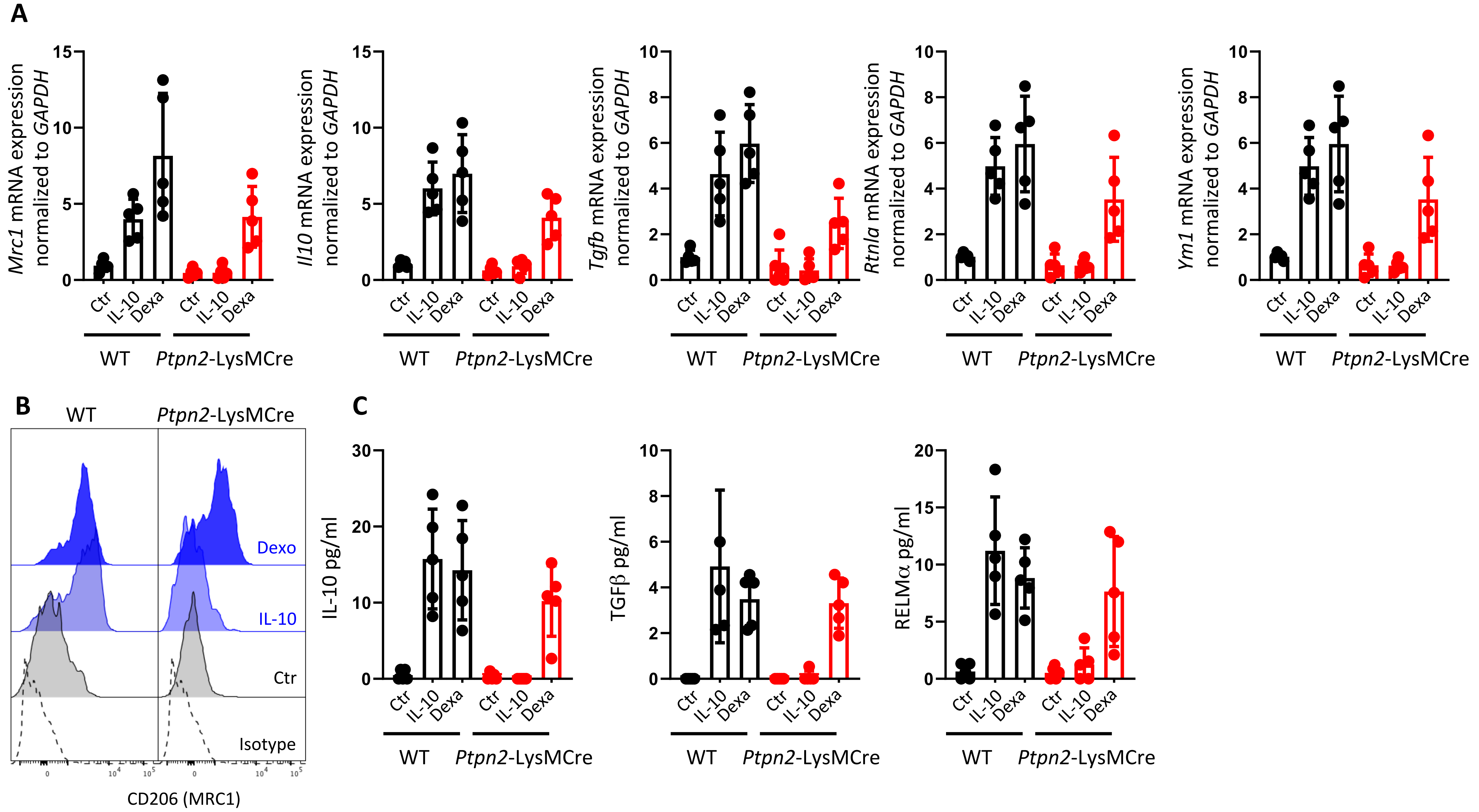

Supplementary Figure S7. PTPN2-deficient macrophages develop into M2 macrophages upon dexamethasone, but not upon IL-10 treatment. BMDM from WT or *Ptpn2*-LysMCre mice were treated for 24 h with IL-10 or dexamethasone and analyzed for **A)** mRNA expression of the indicated genes, **B)** surface expression of CD206, and **C)** secretion of IL-10, TGFb, and RELMa. n = 5 independent repetitions. Related to main Figure 4.

Supplementary Figure S8

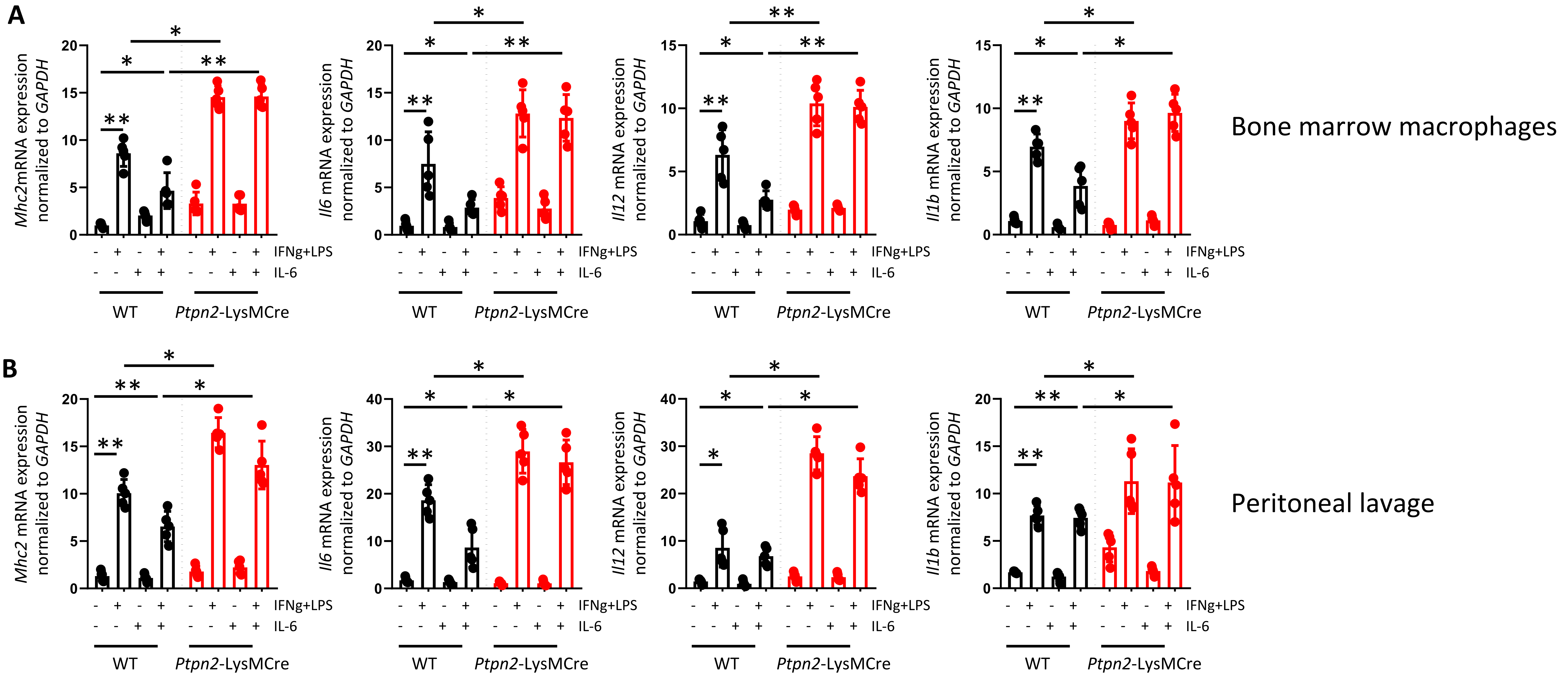

Supplementary Figure S8. Knockdown of PTPN2 promotes M1 marker expression. A) Bone marrow-derived macrophages from WT or *Ptpn2*-LysMCre mice were pre-treated with IL-6 for 6 h prior to treatment with IFNg (500IU) and LPS (50ng) for 24 h and analyzed for mRNA expression of *Mhc2*, *IL6*, *Il12*, and *Il1b*; **B)** *Ptpn2*-LysMCre and their WT littermates were injected intraperitoneally with LPS (1mg) and IFNg (1000IU) and peritoneal cells isolated 24 h later and analyzed as in A. * = $p < 0.05$; ** = $p < 0.01$ relative to untreated control cells, ANOVA with Dunnetts's multiple comparisons test. Pooled data from two independent experiments with 2-3 mice per experiment (n=5).

Supplementary Figure S9

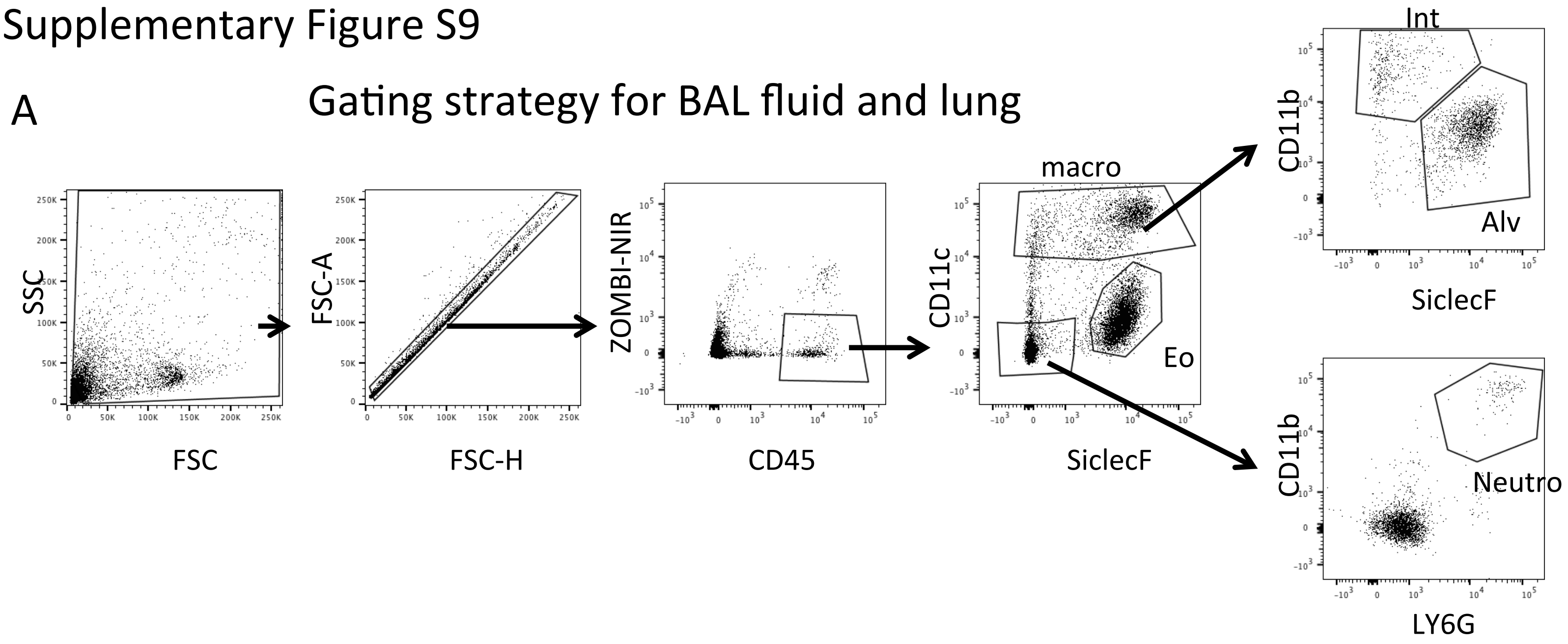

Supplementary Figure S9. Gating strategy for immune cells in the lung and BAL. Representative dot plots and gating strategy used to discriminate different immune cell subsets in the lung and BAL.

Supplementary Figure S10

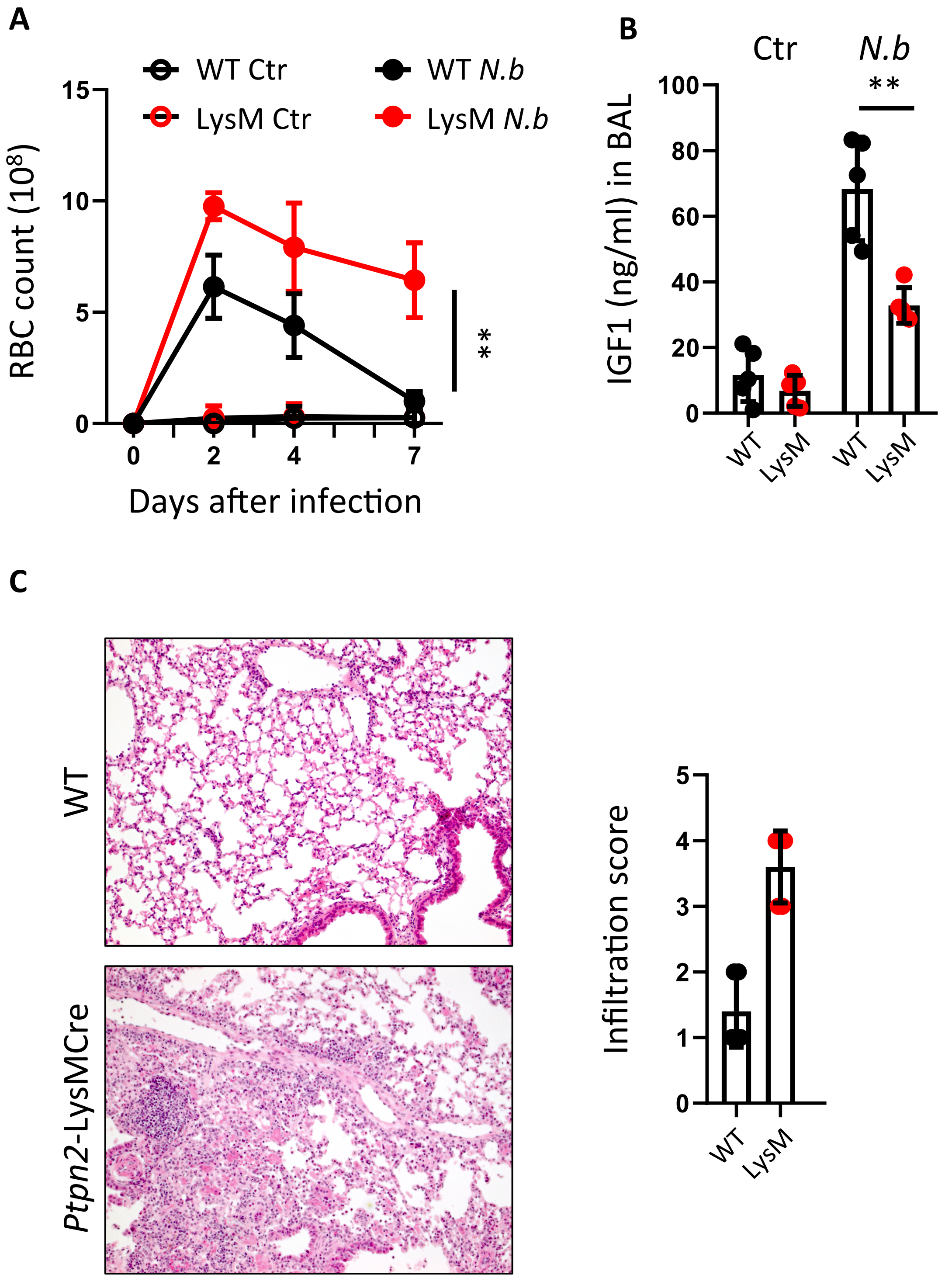

Supplementary Figure S10. Delayed tissue repair in *Ptpn2*-LysMcre mice. WT and *Ptpn2*-LysMCre mice were treated as in Figure 1 and BAL analyzed for **A)** red blood cell count at the indicated time points and **B)** IGF1 at day 7 post infection. **C)** WT or *Ptpn2*-LysMCre mice were infected with 500 L3 larvae, let to recover for 30 days and re-infected with 500 larvae after 30 days. 7 days after the secondary infection lung tissue was harvested and analyzed for lung pathology. n = 5 individual per genotype.

Supplementary Figure S11

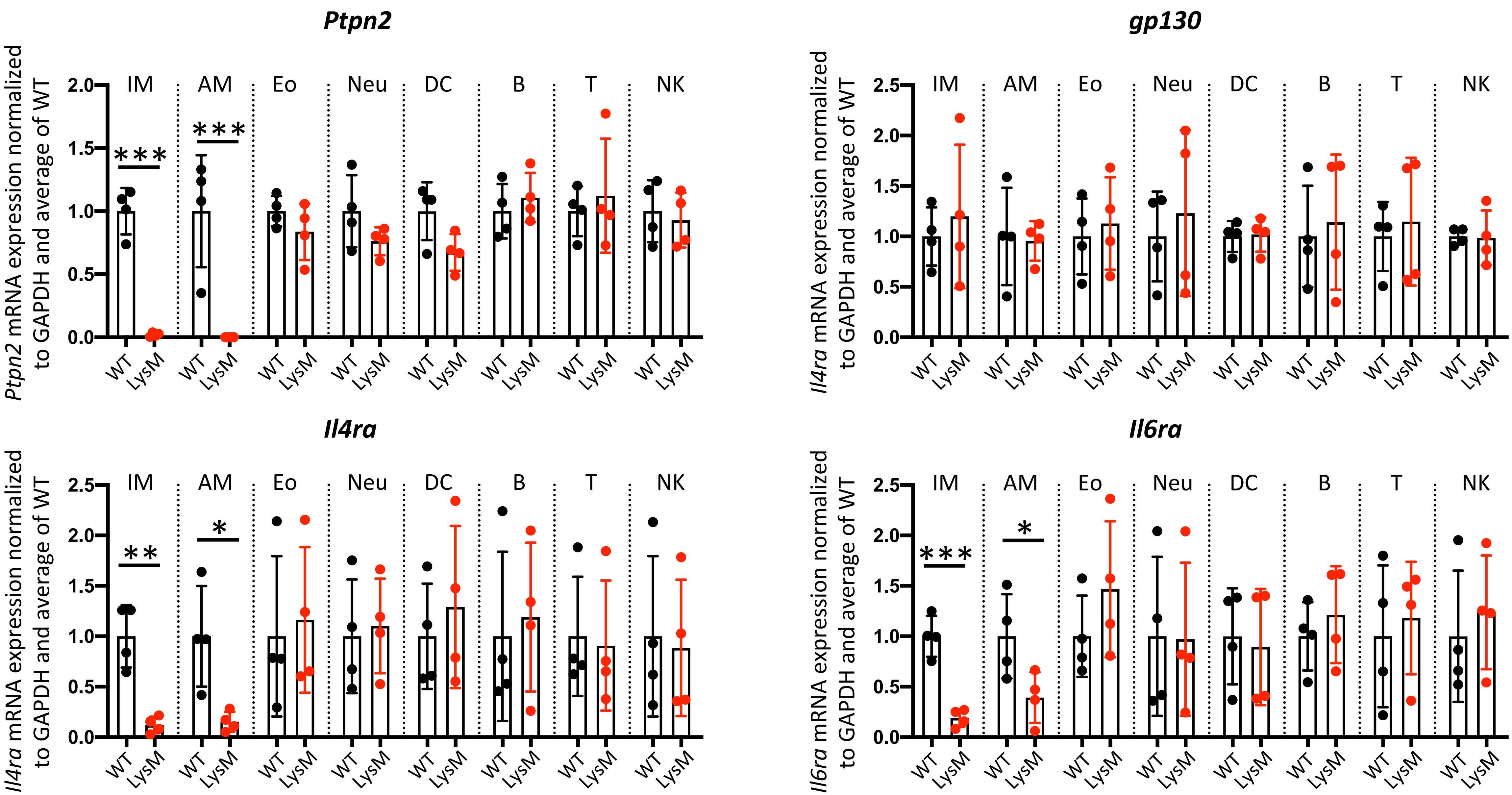

Supplementary Figure S11. Reduced *Il6ra* and *Il4ra* mRNA expression in macrophages from the lung of *Ptpn2*-LysMCre mice. The indicated immune cell subsets were sorted from lung tissue from *N. brasiliensis* infected WT or *Ptpn2*-LysMCre (LysM) mice and analyzed for mRNA expression of *Ptpn2, IL4ra, gp130,* and *Il6ra*. n = 4 mice per genotype. * = $p < 0.05$, ** = $p < 0.01$, Kruskal-Wallis with Dunn's multiple comparisons test. Related to main Figure 7.

Supplementary Figure 12

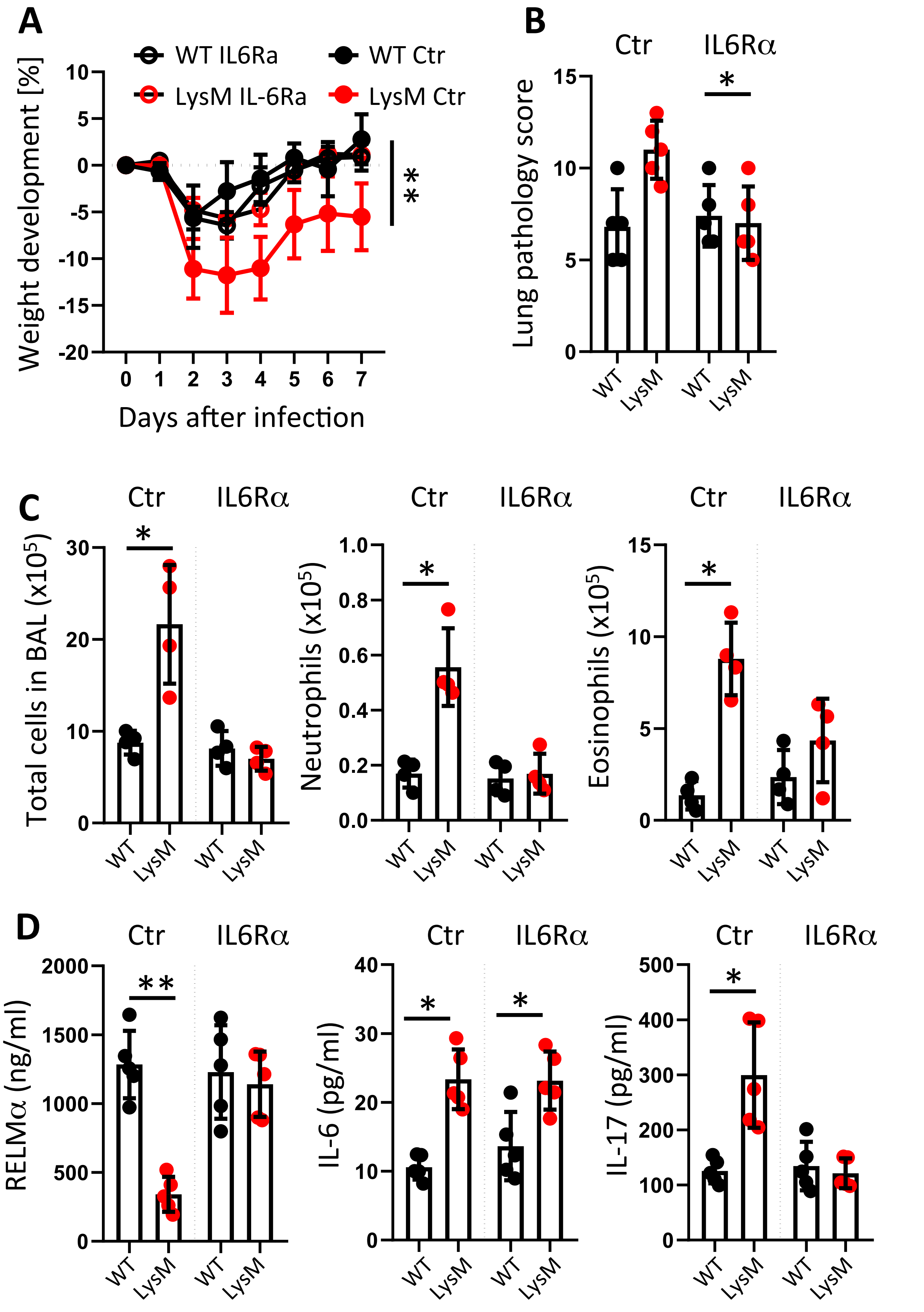

Supplementary Figure S12. IL-6Ra injection reduces severity of *Nippostrongylus brasilensis*-induced disease in *Ptpn2*-LysMCre mice. 7-week-old *Ptpn2*-LysMCre and their WT littermates were infected with 500 *Nippostrongylus brasiliensis* L3 stage larvae and injected with 50mg IL-6Ra intraperitoneally once daily. **A)** Weight development post infection, **B)** lung pathology scores, **C)** numbers of infiltrating cells (total), neutrophils, eosinophils, and macrophages in BAL fluid at day 7. **D)** Levels of the indicated cytokines in BAL fluid on day 7 post infection. Representative results from one out of two independent experiments with 3-5 mice per group, each. * = $p < 0.05$, ** = $p < 0.01$, *** = $p < 0.001$, Kruskal-Wallis with Dunn's multiple comparisons test.
