# Supplementary material for: Loss of protein tyrosine phosphatase non-receptor type 2 reduces IL-4-driven alternative macrophage activation
Source: Mucosal Immunol. 2021 Aug 21;15(1):74–83. doi: 10.1038/s41385-021-00441-3 (PMC8732276; doi:10.1038/s41385-021-00441-3)
Supplement: Supplementary file 2 — Supplementary Table 1 [file 41385_2021_441_MOESM2_ESM.docx]

| **Mouse qPCR primers** | | |
| --- | --- | --- |
| **mGapdh_Fw** | CATCACTGCCACCCAGAAGACTG | |
| **mGapdh_Rv** | ATGCCAGTGAGCTTCCCGTTCAG | |
| **Arg1_Fw** | TGGCTTGCGAGACGTAGAC | |
| **Arg1_Rv** | GCTCAGGTGAATCGGCCTTTT | |
| **mCd206_Fw** | TGATTACGAGCAGTGGAAGC | |
| **mCd206_Rv** | GTTCACCGTAAGCCCAATTT | |
| **Il4ra_Fw** | ACGTGGTACAACCACTTCCAG | |
| **Il4ra_Rv** | CCGCTGTTCTCAGGTGACAT | |
| **mIl10_Fw** | CCCAGAAATCAAGGAGCATT | |
| **mIL10_Rv** | TCACTCTTCACCTGCTCCAC | |
| **mTgfb_Fw** | TGATACGCCTGAGTGGCTGTCT | |
| **mTgfb_Rv** | CACAAGAGCAGTGAGCGCTGAA | |
| **gp130_Fw** | GGCTGGGCGGTCTTTTAGAT | |
| **gp130_Rv** | GAGGTGTCCATTCCACCCAG | |
| **Il6ra_Fw** | GAGACCTGGGACCCGAGTTA | |
| **Il6ra_Rv** | CAAGGAATACGGTGGGGGTG | |
| **Chil3_Rv** | AGAAGGGAGTTTCAAACCTGGT | |
| **Chil3_Fw** | CTCTTGCTGATGTGTGTAAGTGA | |
| **Human qPCR primers** | | |
| **hGAPDH_Fw** | AGATCCCTCCAAAATCAAGTGG | |
| **hGAPDH_Rv** | GGCAGAGATGATGACCCTTTT | |
| **ARG1_Fw** | GGTGGAAGAAGGCCCTACAG | |
| **ARG1_Rv** | TTGCCAAACTGTGGTCTCCG | |
| **CD206_Fw** | CGATCCGACCCTTCCTTGAC | |
| **CD206_Rv** | GATCTGACTCCGGGCATCTG | |
| **hIL4RA_Fw** | CTGCTCATGGATGACGTGGTCA | |
| **hIL4RA_Rv** | GGTGTGAACTGTCAGGTTTCCTG | |
| **hMRC1_Fw** | AGCCAACACCAGCTCCTCAAGA | |
| **hMRC1_Rv** | CAAAACGCTCGCGCATTGTCCA | |
| **hIL10_Fw** | GCTGTAACAGGGACTAGCAC | |
| **hIL10_Rv** | CTGAAGTTAGCAGAGAGCAGG | |
| **hTGFB_Fw** | TACCTGAACCCGTGTTGCTCTC | |
| **hTGFB_Rv** | GTTGCTGAGGTATCGCCAGGAA | |
| **hGP130_Fw** | CACCCTGTATCACAGACTGGCA | |
| **hGP130_Rv** | TTCAGGGCTTCCTGGTCCATCA | |
| **hIL6RA_Fw** | GACTGTGCACTTGCTGGTGGAT | |
| **hIL6RA_Rv** | ACTTCCTCACCAAGAGCACAGC | |
| **hRETN_Fw** | TGGAGTGCCAGAGCGTCACCT | |
| **hRETN_Rv** | ACTGGCAGTGACATGTGGTCTC | |
| **hALOX15_Fw** | ACCTTCCTGCTCGCCTAGTGTT | |
| **hALOX15_Rv** | GGCTACAGAGAATGACGTTGGC | |
| **WB antibodies** | | |
| **Target** | **Supplier** | **Catalogue No** |
| **phospho-STAT3 (Tyr705)** | Cell Signalling Technologies | #9145 |
| **total STAT3** | Cell Signalling Technologies | #12640 |
| **phopho MAK P38 (Thr180/Tyr182)** | Cell Signalling Technologies | #4511 |
| **total MAK P38** | Cell Signalling Technologies | #8690 |
| **phospho SHP2 (Tyr542)** | Cell Signalling Technologies | #3751 |
| **total SHP2** | Cell Signalling Technologies | #3397 |
| **TCPTP** | Merck Millipore | MABS1753 |
| **b-actin** | Cell Signalling Technologies | #3700 |
| **IL-6Ra - mouse** | Cell Signalling Technologies | #39837 |
| **GP130** | Cell Signalling Technologies | #3732 |
| **IL-4Ra** | Thermo Fisher Scientific | #PA5-103142 |
| **IL-6Ra - human** | Cell Signalling Technologies | #12786 |

**Table 1:** Primers used for qPCR and antibodies used for Western blotting
